# Hippocampal cytogenesis and spatial learning in senile rats exposed to chronic variable stress: effects of previous early life exposure to mild stress

**DOI:** 10.3389/fnagi.2015.00159

**Published:** 2015-08-18

**Authors:** Fernando Jauregui-Huerta, Limei Zhang, Griselda Yañez-Delgadillo, Pamela Hernandez-Carrillo, Joaquín García-Estrada, Sonia Luquín

**Affiliations:** ^1^Departamento de Neurociencias, Centro Universitario de Ciencias de la Salud, Universidad de GuadalajaraGuadalajara, Jalisco, Mexico; ^2^Departamento de Fisiología, Facultad de Medicina, Universidad Nacional Autónoma de MéxicoMéxico, Mexico; ^3^División de Neurociencias, Centro de Investigación Biomédica de Occidente (CIBO), Instituto Mexicano del Seguro SocialGuadalajara, Mexico

**Keywords:** glia, noise, dentate gyrus, aging, astrocytes, memory

## Abstract

In this study, we exposed adult rats to chronic variable stress (CVS) and tested the hypothesis that previous early-life exposure to stress changes the manner in which older subjects respond to aversive conditions. To this end, we analyzed the cytogenic changes in the hippocampus and hippocampal-dependent spatial learning performance. The experiments were performed on 18-month-old male rats divided into four groups as follows: *Control* (old rats under standard laboratory conditions), *Early-life stress* (ELS; old rats who were exposed to environmental noise from postnatal days, PNDs 21–35), CVS + ELS (old rats exposed to a chronic stress protocol who were previously exposed to the early-life noise stress) and CVS (old rats who were exposed only to the chronic stress protocol). The Morris Water Maze (MWM) was employed to evaluate the spatial learning abilities of the rats at the end of the experiment. Immunohistochemistry against 5′Bromodeoxyuridine (BrdU) and glial fibrillar acidic protein (GFAP) was also conducted in the DG, CA1, CA2 and CA3 regions of the hippocampus. We confocally analyzed the cytogenic (BrdU-labeled cells) and astrogenic (BrdU + GFAP-labeled cells) changes produced by these conditions. Using this procedure, we found that stress diminished the total number of BrdU+ cells over the main proliferative area of the hippocampus (i.e., the dentate gyrus, DG) but increased the astrocyte phenotypes (GFAP + BrdU). The depleted BrdU+ cells were restored when the senile rats also experienced stress at the early stages of life. The MWM assessment demonstrated that stress also impairs the ability of the rats to learn the task. This impairment was not present when the stressful experience was preceded by the early-life exposure. Thus, our results support the idea that previous exposure to mild stressing agents may have beneficial effects on aged subjects.

## Introduction

Aging and stress are conditions that compromise the adaptive capabilities of all organisms. The brain structures mediating highly adaptive capabilities become considerably affected both when subjects are exposed to chronic/intense stress and when subjects reach advanced stages of life (Miller and O’Callaghan, 2003). The hippocampus has emerged as the most studied brain structure for both pathological aging and the deregulated stress responses (Sapolsky et al., 1986). The relevance of the hippocampal circuits to pathological aging arises from their recognition as the main anatomical substrate behind the cognitive decline that characterizes senescence (Samson and Barnes, 2013). The hippocampal regions are also major regulators of stress responses and frequently lose their regulatory skills under extreme conditions (Kim et al., 2006).

The hippocampus is a well-described limbic region that serves as the physical background for the acquisition/consolidation of spatial memory (O’Keefe and Dostrovsky, 1971). It also conserves its cytogenic capabilities for practically all of the neural lineages (Eriksson et al., 1998). Consistently, studies on deregulated stress responses and pathological aging have described cytological alterations and memory impairments involving the hippocampal formation (Gould and Tanapat, 1999; Karten et al., 2005; Dranovsky and Hen, 2006; Thuret et al., 2009). The proliferative changes that accompany stress have been primarily attended under neurogenic perspectives. Studies on neurogenesis have demonstrated that the proliferative rates may vary in two possible ways: (i) inhibiting the proliferation, survival or differentiation of specific lineages; or (ii) stimulating one or more of those parameters. The results of these studies suggest that the inhibitory/stimulatory effects of stress depend on a series of factors, including the subject’s age, cellular lineage, type and history of stress, etc. (Karten et al., 2005; Dranovsky and Hen, 2006; Thomas et al., 2007).

The starring role of neurons in hippocampal cytogenesis has been ameliorated by the recent recognition of glial cells as the main regulators of processes involved in the proliferation, differentiation and survival of newborn cells (Song et al., 2002; Jauregui-Huerta et al., 2010b; Ekdahl, 2012). In addition to neurons, glial cells have emerged as promising new players whose actions could explain many of the impairing effects of stress and aging in the hippocampus. Glial cells provide support for neurons and control most of the adaptive/regenerative actions, including inflammatory responses (Fields and Stevens-Graham, 2002; Garcia-Segura and McCarthy, 2004). They respond to damage by changing their morphology and proliferation rates to protect and/or reestablish parenchymal homeostasis (Pekny and Nilsson, 2005; Robel et al., 2011). Of the glial cells, astrocytes represent the most versatile population. Their role in stress pathology has been demonstrated in postmortem studies showing that the prefrontal cortices of chronically stressed subjects exhibit reduced numbers of astrocytes (Banasr et al., 2010; Smiałowska et al., 2013). Their relevance in the hippocampal function was also demonstrated in experiments showing that rats with impaired spatial memory possessed fewer astrocytes (Jahanshahi et al., 2008). While it seems clear that aging is accompanied by increased numbers of astrocytes (Lee and MacLean, 2015), it remains controversial whether stress exerts the classical glucocorticoid-mediated anti-proliferative actions as observed in other organs or favors a more pro-inflammatory-like response, similar to other damaging conditions in the brain.

It has been proposed that the effects of stress on organisms may depend on their history of exposure (Bartolomucci et al., 2005; Tsoory et al., 2008; Jauregui-Huerta et al., 2010a; Pastor-Ciurana et al., 2014). The effects of stress on hippocampal functions should not be expected to be the same in younger subjects, whose regulatory systems are immature, as in older subjects, whose adaptive capabilities are compromised. The evidence from experiments on the stress-aging relationship have documented one maladaptive scenario, where aging acts as an undermining factor for the damaging mediators of stress (Sapolsky et al., 1986; Miller and O’Callaghan, 2003; Lavretsky and Newhouse, 2012; Maggio et al., 2013), and other more adaptive scenes, where stress experiences actually strength the homeostatic regulatory systems of aged subjects (Minois, 2000; Calabrese and Baldwin, 2003; Cornelius et al., 2013). Thus, the history of stress may be an important condition that influences the effects on the aged hippocampus. The experiences in the early stages of life seem to be particularly relevant (Welberg and Seckl, 2001; Shors, 2006; Tang et al., 2006; Tsoory et al., 2008; Weinstock, 2008). Because early-life experiences determine the way in which the adult subjects respond to stress, it follows that the hippocampal reactions should be different depending on whether the individuals were exposed to stress at the early stages of life.

Therefore, in this experiment, we evaluated the effects of stress on the hippocampus of senile male rats, and tested the hypothesis that early-life exposure to stress changes the manner in which older subjects respond to aversive conditions. The Morris Water Maze (MWM) was selected to evaluate the main cognitive function of the hippocampus (spatial learning), and the 5′Bromodeoxiuridine (BrdU) + glial fibrillar acidic protein (GFAP) immunostaining method was conducted to estimate the corresponding proliferative/gliogenic changes in this tissue.

## Materials and Methods

### Animals

The subjects were 40 Swiss Wistar male rats obtained from an in-house breeding facility at the Centro de Investigacion Biomedica de Occidente (CIBO), Instituto Mexicano del Seguro Social (IMSS), Guadalajara, Mexico. The rats were weaned on postnatal day 21 (PND 21), housed in standard polycarbonate cages and maintained on a 12 h light-dark cycle, with the lights on at 07:00. Standard Purina rat chow pellets and tap water were provided *ad libitum*. The experimental procedures were approved by the institutional ethics commission and were in accordance with the US National Institute of Health Guide for the Care and Use of Laboratory Animals.

**Figure 1:**
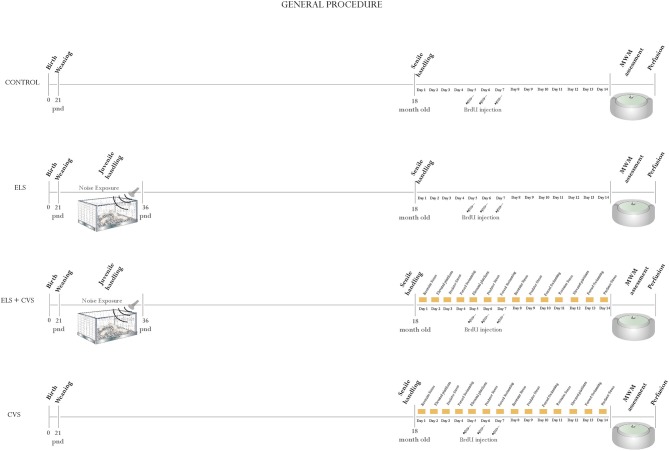
**General procedure: Illustrates the general procedure followed in our experiment for the **(A)** Control group, **(B)** Early-life stress (ELS) group, **(C)** Early-life stress + Chronic variable stress (ELS + CVS) group, and **(D)** Chronic variable stress (CVS) group**. The experimental procedures are chronologically depicted above and below the line. The postnatal age is written below the line.

At the age of 21 PNDs, the rats were assigned to one of four conditions as follows (Figure 1 illustrates the general procedure):

1. *Control*: 10 rats that remained undisturbed until the age of 18 months, when they were evaluated and sacrificed.
2. *Early-life stress (ELS)*: 10 rats that were exposed to environmental noise from PNDs 21–35 and then evaluated and sacrificed 18 months later.
3. *ELS + Chronic variable stress (CVS)*: 10 rats that were exposed to environmental noise from PNDs 21–35 and then re-exposed to a CVS protocol at the age of 18 months.
4. *CVS*: 10 rats that remain undisturbed until the age of 18 months, when they were exposed to a CVS protocol.

### Procedures

#### Early-Life Stress (from PND 21 to PND 35)

For exposure to mild stress at the early stages of life, the rats were exposed to an audiogram-fitted noisy environment (kindly provided by Dr. A. Rabat). Noise is a known ubiquitous stimulus that is capable of inducing a moderate stress response (Rabat et al., 2004; Jauregui-Huerta et al., 2010a; Jáuregui-Huerta et al., 2011). Urban audio files containing unpredictable noise events, with a duration ranging from 18 to 39 s and spaced by silent intervals ranging from 20–165 s, were randomly presented to rats for 24 h throughout the 15 days following weaning (i.e., PNDs 21–35). The animals were housed in a special sound-isolated acoustic stress chamber equipped with professional tweeters (Steren 80–1088) that were suspended 60 cm above the solid grid cages and connected to an amplifier (Mackie M1400; freq. 20 Hz–70 kHz; 300 W-8 Ω) with mixer software that delivered the acoustic signal at levels ranging from 70 dB for the background noise to 85–103 dB for the noisy events. To make sure that the sound intensity was homogeneous at all places in the cage, the noise intensity was measured by placing a sound-level meter (Radio Shack, Mexico; Bohbot et al., 1998).

#### Late-Life Stress (18-Month-Old-Rats)

To induce stress in senile rats, we adapted a standardized protocol that has been previously proven to be efficient in impairing proliferation in adult rats (Banasr et al., 2007). The CVS model administered to aged rats consisted of exposure to four different stressors (restraint-overcrowding, elevated platform, predator stress and forced swim) following the schedule indicated in Figure 1. The animals were exposed to CVS for 14 days in a different room. For restraint-overcrowding, the rats were placed in a small cage for 6 h, where the available space per rat was not enough to permit free movement. For the elevated platform, unavoidable stress was evoked by placing the rat on an elevated platform (21 × 20 cm^2^, located 90 cm above the ground) in the middle of a brightly lit room for 30 min. For the forced swim, the rats were placed into transparent cylindrical plastic tanks (height = 45 cm, internal diameter = 19 cm) containing water (25°C) to a level of 28 cm, where they remained for 15 min. For predator stress, the animals were placed individually in a small mesh cage (50 × 35 × 30 cm) and then introduced to the stress room where an adult female cat had previously been placed. The cat was from domestic origin and the predator experience (odor, appearance and sound but avoiding physical contact) lasted 15 min/day.

#### BrdU Injection

To label newly born cells in the hippocampus, the thymidine analog BrdU (Sigma-Aldrich, St. Louis, MO, USA) was administered to aged rats 10 days before they were sacrificed. All animals received a total of three i.p. injections of BrdU (50 mg/kg, dissolved in 0.9% NaCl) on days 5, 6 and 7 of the late-stress exposure (see Figure 1).

#### Morris Water Maze (MWM)

Once the animals completed the late-life stress protocol, spatial learning was evaluated using the MWM. Because classical MWM trainings have been shown to affect hippocampal cytogenesis (Epp et al., 2011), a short 8-trial training paradigm was employed to avoid additional confounds. The conventional black circular pool (180 cm in diameter, 40 cm height) was filled with warm water (23 ± 3°C) to a depth of 32 cm. A circular black escape platform (10 cm diameter × 30 cm high) was submerged, so that it remained hidden from sight. Spatial cues were placed around the pool, which the rat could use to navigate the maze. Eight trials per animal were executed on the same day as previously described (Hernandez et al., 2012). To begin a trial, the rat was placed in the water and allowed to swim until it found the hidden platform. If the rat did not find the platform within the allotted time (60 s, it was gently guided to the platform. Once on the platform, the rats were permitted to stay there for 15 s. The test consisted of eight consecutive trials with 5 min rest intervals that were video-recorded. An off-line blinded analysis was performed to analyze the escape latency (time to reach the escape platform). No probe trial was conducted because the animals were sacrificed immediately after the 8th trial. We avoided with this procedure the objectionable effects of training on cytogenesis and the undesirable effects of excessive swimming on the fragile health state of senile rats.

#### Immunohistochemical Analyses

Once the animals completed their corresponding stress and training procedures, the animals (5 per group) were transcardially perfused using a standard protocol (Hernández et al., 2014). We performed a double immunofluorescence staining method to confocally co-localize cells that went through mitosis (BrdU+) with the filamentary protein expressed throughout the cytoskeleton of astrocytes, glial fibrillar acidic protein (GFAP; astrogenesis), in the hippocampal dentate gyrus (DG) and cornus ammonis (CA1, CA2, CA3) regions.

A series of systematically selected brain sections (40 μm-thick, every 200 μm from bregma 2.1 to −4.5 mm) were manually counted using the 20× objective for the BrdU+ cells on left and right hippocampus, and the 4× objective for the BrdU + GFAP+ cells. The mean number of BrdU+ cells per microscopic field (left and right hippocampus on 12 sections per animal) was reported as cytogenesis, while the BrdU+ cells that co-localize with GFAP+ was reported as astrogenesis. We considered that BrdU and GFAP co-localized when the green nuclear fluorescence (BrdU) coincided with red somal fluorescence (GFAP) in consecutive 1 μm z-stacks and when the co-localization was confirmed in x-y, x-z, and y-z cross-sections. We assessed the changes in BrdU + GFAP staining using a confocal laser-scanning microscope (Leica TCS SP2) connected to a PC with the Leica confocal software (LCS). For every section, the percentage of co-localized BrdU+ cells was calculated as the fraction of the number of BrdU+ cells that co-expressed GFAP/the total number of BrdU+ cells per section multiplied by 100.

The immunofluorescence protocols were conducted using a series of free-floating sections from bregma 2.1 to −4.5 mm (Paxinos and Watson, 1982) which were first pre-treated to denature the DNA. The sections were incubated in 2N HCl for 30 min at 37°C and rinsed with 0.1 M borate buffer (pH 8.5). Then, the slices were washed with phosphate buffer (PB) and incubated with blocking solution (10% normal goat serum in 0.1 PB) for 60 min at room temperature. The tissues were then incubated for 12 h at 4°C with a monoclonal antibody against BrdU (rat anti-BrdU, Accurate Scientific, OBT003, dilution 1:1000) and a polyclonal antibody against GFAP (rabbit anti-GFAP, Dako, dilution 1:500). After rinsing in PB, the sections were incubated for 2 h with the secondary antibodies (Alexa Fluor 488 goat anti-rat IgG and Alexa Fluor 594 goat anti-rabbit from Molecular Probes at 1:1000 dilutions). The sections were mounted on slides and cover-slipped using fluorescent mounting media (Vectashield Vector Labs, Burlingame, CA, USA).

#### Statistical Analysis

The statistical analyses were performed using Excel (Microsoft Office for Mac 2011) and IBM SPSS statistics version 20. The quantitative results were expressed as the means ± standard error of mean (SEM). Analysis of variance ANOVA was employed to compare escape latencies and to analyze the BrdU/GFAP-labeled cells. Tukey-Scheffé *post hoc* tests were used for subsequent comparisons. The differences were considered statistically significant at *p* < 0.05 (*p < 0.05, **p < 0.01, and ***p < 0.001).

## Results

### Effect of Stressing Conditions on Spatial Learning

**Figure 2:**
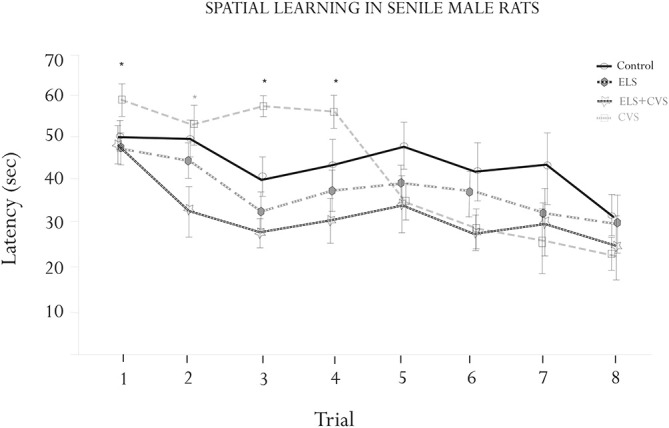
**Morris water maze (MWM) assessment**. Figure shows the results of the escape latencies across trials (means ± SEM). Significant interactions were found for the escape latency for trials 1, 3 and 4 (*p < 0.05), where the ELS-exposed animals showed increased latencies. A significant interaction was found for trial 2, where the ELS + CVS-exposed animals decreased the latency to find the hidden platform (*p < 0.05). The ELS-exposed rats were no different than the control rats.

The effects of the different stress conditions on spatial learning were tested using the hidden-platform water maze paradigm (MWM). The motivational behavior to swim was evaluated by measuring the swim speed between groups. We did not find differences in swimming ability or motivation, as the swim speed was equal between groups (data not shown). Figure 2 illustrates the MWM results.

ANOVA demonstrated differences between groups to find the platform at specific trials corresponding to the first half of the assessment. Tukey *Post hoc* analysis revealed that senile rats exposed to CVS took a longer time to reach the platform than did those of the other three groups. The differences were statistically significant on trials 1 (*F*_(1,17)_ = 7.079, *p < 0.05), 3 (*F*_(1,17)_ = 7.336, *p < 0.05), and 4 (*F*_(1,17)_ = 8.173, *p < 0.05). No differences were found from trial 5–8, indicating that the CVS rats completed the training under the normal parameters.

The doubly stressed rats (ELS + CVS) tended to find the hidden platform faster than the other groups; however, this trend reached significance only in trial 2 compared to all other groups (*F*_(1,17)_ = 10.118, *p < 0.05).

Finally, the senile rats that only received stress at early stages of life (ELS) did not demonstrate any differences in the MWM assessment, suggesting that they had recovered their ability to perform this task.

### Effect of Stress Conditions on the BrdU-Labeled Cells (Hippocampal Cytogenesis)

To determine whether stress conditions modify hippocampal cytogenesis, we quantified the number of BrdU immunopositive cells per microscopic area (0.750 mm^2^) in the DG, CA1, CA2 and CA3 regions.

**Figure 3:**
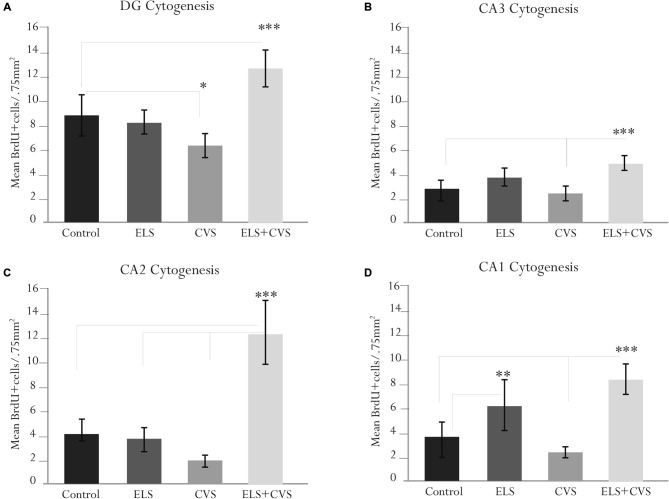
**Hippocampal cytogenesis in senile male rats**. The figure contains graphics illustrating the means ± SEM of the BrdU-positive cells in the **(A)** dentate gyrus (DG), **(B)** cornus ammonis (CA3), **(C)** CA2, and **(D)** CA1 subregions. The ELS + CVS-exposed rats exhibited an increased number of BrdU+ cells in the DG (***p < 0.001), CA3 (***p < 0.001), CA2 (***p < 0.001), and CA1 (***p < 0.001). The CVS-exposed rats exhibited a decreased number of 5′Bromodeoxyuridine (BrdU+) cells in the DG (*p < 0.05). The ELS-exposed rats exhibited an increased number of BrdU+ cells in the CA1 region (**p < 0.01).

First, we investigated whether stress conditions modified the proliferation in the DG area, whose subgranular zone has been recognized as the main proliferative region of the hippocampus. ANOVA analysis of this area revealed a significant difference between groups, *F*_(12,309)_ = 10.98, ***p < 0.001, with doubly stressed rats (ELS + CVS) scoring higher (*M* = 12.65, ***p < 0.001) and chronically stressed rats (CVS) scoring lower (*M* = 6.18, *p < 0.039) than the controls (*M* = 8.7; Tukey *post hoc* test). No significant changes were observed in the ELS group compared to the control rats (Figure 3A).

Next, we determined the effects of stress conditions on the CA3 area. Again, ANOVA revealed a significant difference between groups in this area, *F*_(12,309)_ = 8.78, ***p < 0.001. *Post hoc* analysis demonstrated that the ELS + CVS rats scored higher (*M* = 5.1) than the control (*M* = 2.87, ***p < 0.001) and CVS rats (*M* = 2.0, ***p < 0.001). No significant changes were observed in this region for the CVS or ELS groups compared to the controls (Figure 3B).

Next, we quantified the BrdU+ cells in the CA2 area. Quantification of the proliferative cells in the CA2 region also demonstrated differences between groups, *F*_(12,309)_ = 14.41, ***p < 0.001. Once again, the CVS + ELS rats showed the highest numbers (*M* = 8.64) compared to the control (*M* = 4.33, ***p < 0.001), ELS (*M* = 4.30, ***p < 0.001), and CVS rats (*M* = 2.81, ***p < 0.001; Figure 3C).

Finally, the CA1 area also demonstrated significant differences between groups, *F*_(12,309)_ = 15.40, ***p < 0.001. Rats belonging to the ELS + CVS group increased their proliferation (*M* = 8.88) in relation to the control (*M* = 3.63, ***p < 0.001) and CVS groups (*M* = 3.00, ***p < 0.001). Exceptionally, the ELS rats increased the number of BrdU+ cells (*M* = 6.65) in this area compared to the control (*M* = 3.63, ***p < 0.001) and CVS rats (*M* = 3.00, ***p < 0.001; Figure 3D).

### Effect of Stress Conditions on the BrdU + GFAP Co-Labeled Cells (Hippocampal Gliogenesis)

**Figure 4:**
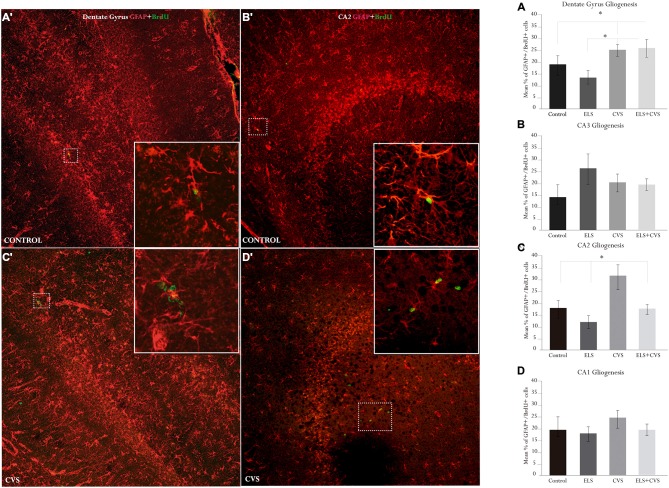
**Hippocampal gliogenesis in senile male rats**. The right panel contains graphics illustrating the means ± SEM percentage of glial fibrillar acidic protein (GFAP) + BrdU-positive cells in the **(A)** dentate gyrus, **(B)** CA3, **(C)** CA2, and **(D)** CA1 subregions. The ELS + CVS-exposed rats exhibited an increased number of GFAP + BrdU+ cells in the DG (*p < 0.05). The CVS-exposed rats exhibited an increased percentage of GFAP + BrdU-positive cells in the CA2 region (*p < 0.05). The ELS-exposed rats exhibited an increase in the numbers of GFAP + BrdU-positive cells in the CA3 region (*p < 0.05). The left panel illustrates co-labeling in the two statistically relevant regions: **(A′)** DG, and **(B′)** CA2 (slices 730 μm × 730 μm). The white boxes in **(C′)** and **(D′)** illustrate co-labeling; the round green cells are BrdU+, and the star-shaped red cells are GFAP+.

By sacrificing the rat 10 days after the first BrdU injection, we were also able to determine the glial phenotype of the new cells that incorporated BrdU. We used the glial marker GFAP to estimate the percentage of newly generated cells (i.e., BrdU+) that correspond to astrocytes. Figure 4 illustrates the hippocampal gliogenesis.

First, we determined the effects of stress conditions on astrocyte proliferation in the DG by counting the proportion of BrdU+ cells that co-expressed GFAP. ANOVA analysis revealed that exposure to stress at senescence stimulates the proliferation of astrocytes in the main proliferating area of the hippocampus. We found significant differences between groups for this area, *F*_(12,243)_ = 2.555, *p < 0.05. *Post hoc* analyses revealed that the proportion of double-labeled cells was increased in both the ELS + CVS (*M* = 24.96) rats and the CVS (*M* = 24.84) rats compared to the control (*M* = 19.0, *p < 0.05) and ELS rats (*M* = 14.2, *p < 0.05; Figure 4A).

Next, we evaluated the effects of the experimental conditions on gliogenic activity outside the main hippocampal cytogenic area. We found that stress selectively increased the number of newborn astrocytes in the CA2 area, as group differences were only evident in this area, *F*_(12,243)_ = 3.622, *p < 0.05. Here, the CVS rats had the highest proportion of new astrocytes (*M* = 32.36) compared to the Control (*M* = 18.70, *p < 0.05), ELS (*M* = 12.73, *p < 0.01), and ELS + CVS rats (*M* = 16.54, *p < 0.05; Figures 4B–D).

## Discussion

In this study, we evaluated the effects of CVS on hippocampal cytogenesis and spatial learning in senile rats. We also tested the hypothesis that the effects of stress on these parameters are determined by early-life experiences. We found that stress diminished the total number of BrdU+ cells in the main proliferative area of the hippocampus (i.e., the DG), but increased the number of newborn astrocytes (GFAP + BrdU). The decreased number of BrdU+ cells was reversed when the senile rats also experienced stress at the early stages of life. The results from the MWM assessment demonstrated that stress impaired the task acquisition. This impairment was abolished when the stressful experience was preceded by the early-life exposure.

The effects of stress on cellular proliferation have been previously investigated. Previous experiments have shown that chronic or intense conditions reduce the number of newborn cells in the DG and other neurogenic structures (Malberg and Duman, 2003; Karten et al., 2005; Czéh et al., 2007; Thomas et al., 2007). Neurons (i.e., neurogenesis) have been studied the most. Here, we confirmed that exposure to intense stress affects the hippocampus through the classical anti-proliferative pathway previously reported for other intense or chronic conditions. It seems that senile rats also adjust to the anti-inflammatory hypothesis, which claims that stress mediators suppress proliferation (BrdU+ counts). Accordingly, other studies reported inhibition of cell proliferation under similar conditions (Lemaire et al., 2000; Yan et al., 2011; Gil-Mohapel et al., 2013).

Conversely, quantification of the cytogenic profiles in the doubly stressed rats (ELS + CVS) demonstrated that the reduced hippocampal proliferation was reversed. We found increased numbers of BrdU+ cells in those who experienced stress at both stages of life. It is known that some physiological and/or pathological conditions may stimulate proliferation in adult neurogenic and non-neurogenic structures. Physical activity, anti-depressants, ischemia, trauma and other conditions increase the number of proliferative cells in the hippocampus and other brain regions (Gonzalez-Perez et al., 2010; Lyons et al., 2010; Parihar et al., 2011; Suri et al., 2013). The stimulatory effects of stress on proliferation may be expected because the pro-inflammatory actions of stress mediators were documented both at the early and late phases of the stress response (Sorrells and Sapolsky, 2007). Support for results obtained in this part of our study may be extracted from studies showing that chronic exposure to mild and/or predictable stress enhances hippocampal neurogenesis (Lyons et al., 2010; Parihar et al., 2011); complementary experiments demonstrated that the long-term survival of these newborn cells increased if the pups were subjected to chronic stress (Chocyk et al., 2015). Moreover, outstanding support for these findings has been provided by elegant experiments showing that neurogenesis was improved when young adult rats exposed to severe stress also received stress at the early stages of life (Oomen et al., 2010). Thus, viewed in this context, our results support the hypothesis that early-life experiences may influence cytogenic processes during later life. In addition to the age-limited ranges analyzed in the above-mentioned experiments, we extended these results to senescence and confirmed that new aversive experiences may serve as a trigger for these stimulating effects.

The results obtained from the BrdU + GFAP counts also demonstrated the stimulating effects of stress on hippocampal gliogenesis. We found that stress promoted astrocyte proliferation in senile subjects, which was opposite to the expected results under the suppressive view. This effect contrasted with the observations of the single-labeled cells (BrdU+), indicating a phenotypic difference. The phenotypic differences in stress responsiveness have been previously demonstrated for younger rats (Gonzalez-Perez et al., 2011). Confocal analyses conducted in the cerebral cortex showed that neurons, oligodendrocytes and endothelial cells differentially changed their proliferative rate under stress conditions (Banasr et al., 2007). Our results confirmed that chronic stress affects hippocampal proliferation in a phenotype-specific manner and introduced evidence suggesting that astrocytes proliferate even when other lineages are impaired. There are only a few studies analyzing hippocampal astrocytes under stress conditions. Most of them reported the total number of astrocytes and their results are controversial. While in some cases stress increased the number of astrocytes (Lambert et al., 2000), in other cases, their total numbers were decreased (Czéh et al., 2006; Han et al., 2015). Because our analyses included proliferation (GFAP + BrdU) and not just the total numbers (GFAP+), the data should serve as cumulative evidence that considers senescence as a favorable condition for stress-induced proliferation of glial cells. Consequently, the increased astrogenesis may explain the increased number of astrocytes reported in the above-mentioned experiments. Because increased numbers of astrocytes have been reported in pathological aging and other damaging conditions (Lee and MacLean, 2015), it may be argued that the increased gliogenesis described in this study could adjust to the “reactive gliosis” profile reported for these conditions. It seems to be the case for the increased astrogenesis in the CA, a non-classical proliferative subregion. However, the increased numbers of BrdU + GFAP-positive cells in then DG could be viewed in a different context. Because subgranular astrocytes have been shown to play crucial roles in neuron renewal, the changes in proliferative activity could be physiologically beneficial. In accord with this inference, previous experiments showed that corticosterone treatment (the main stress effector) increased astrocyte numbers (Bridges et al., 2008) and coincidently, spatial learning (Jahanshahi et al., 2008). Nevertheless, the functional implications of the augmented gliogenesis following stress in senile rats should be addressed in future experiments.

The results obtained from the MWM assessment resembled a similar pattern as the immunohistochemical results. While the rats exposed to chronic stress at senescence exhibited poorer performance, the performance of rats that also received stress as juveniles was normal. Thus, even when the stress-induced cognitive impairments were not as dramatic as in other experiments; it is noticeable that the CVS rats increased their latencies during the first half of the assessment, indicating a change in swimming strategies that ultimately disrupted the expected performance (Gil-Mohapel et al., 2013). The stress-induced impairments have been frequently demonstrated in young or adult rats, but only a few have evaluated senile rats (Lupien et al., 1998; Sandi and Touyarot, 2006; Borcel et al., 2008). Coincidently, those studies also investigated the cognitive changes produced by previous exposures to stress. Because the results of these experiments differ from our results by showing that senile performance was impaired by stress administered at adulthood, it is arguable that the stress onset (early life vs. adulthood) plays a central role in directing the long-lasting effects on cognition. On the other hand, it has been proposed that both escape latencies and acquisition strategies are related to hippocampal cytogenesis (Garthe et al., 2009). For the most part, the decreased number of BrdU+ cells in the DG correlates with impaired spatial learning and *vice versa* (Lemaire et al., 2000; Aisa et al., 2007; Wu et al., 2007; Thuret et al., 2009; Gil-Mohapel et al., 2013). There is a substantial coincidence between this view and the data showing that conditions that reduced proliferation (i.e., CVS) also affected acquisition in our MWM assessment. There is a plethora of experiments showing that intense stress impairs both neurogenesis and spatial learning (Lemaire et al., 2000; Sandi et al., 2005; Yan et al., 2011; Naninck et al., 2015). It seems clear that intense and/or chronic conditions impair these parameters at all stages of life and that such effects are reversible, as shown in our experiments. There are only a few experiments addressing conditions where stress stimulates proliferation and cognition. Previous evidence (Oomen et al., 2010) showed that severe ELS inhibits neurogenesis and spatial learning, but improves hippocampal function under high-stress conditions in adulthood, supporting the stimulating/adaptive profile of our results. If that is the case, our results extend to the later part of life (i.e., senescence), and support the body of evidence indicating that early-life aversive experiences may prepare organisms to respond adaptively to threatening conditions later in life. It seems that the benefits of these otherwise maladaptive responses may become relevant in times of crisis, as is the case of new stressful conditions and/or senescence.

In conclusion, our results confirmed that chronic stress negatively affected hippocampal proliferation and spatial learning in senile rats. Nevertheless, we demonstrated that these inhibitory effects may be reversed when the senile rats also experienced stress at the early stages of life. We also provide evidence that chronic stress may stimulate astrocyte proliferation in the hippocampus of senile subjects. Taken together, these results support the notion that early experiences may extend their cellular and/or cognitive sequelae into senescence and provide evidence that glial cells should be further considered in studies addressing the stress-aging relationship.

## Author Contributions

JG-E, SL, LZ, and FJ-H conceived and designed the experiments. GY-D, PH-C, and FJ-H performed the experiments. LZ, GY-D, PH-C, and FJ-H analyzed the data. LZ, JG-E, SL, and FJ-H contributed reagents, materials, and analysis tools. FJ-H wrote the paper.

## Funding

This work was supported by the following research grants: CONACyT 221092, CONACyT 238313, PAPIIT-UNAM 216214 and PRODEP red temática UDG CA63-UCOL CA05.

## Conflict of Interest Statement

The authors declare that the research was conducted in the absence of any commercial or financial relationships that could be construed as a potential conflict of interest.
